# A Modeling Approach for Quantifying Human-Beneficial Terpene Emission in the Forest: A Pilot Study Applying to a Recreational Forest in South Korea

**DOI:** 10.3390/ijerph19148278

**Published:** 2022-07-06

**Authors:** Kwanghun Choi, Dongwook W. Ko, Ki Weon Kim, Man Yong Shin

**Affiliations:** Department of Forest, Environment and Systems, Kookmin University, Seoul 02707, Korea; kchoi@kookmin.ac.kr (K.C.); kwkim@kookmin.ac.kr (K.W.K.); yong@kookmin.ac.kr (M.Y.S.)

**Keywords:** forest healing, terpenes, Model of Emissions of Gases and Aerosols, biogenic volatile organic compounds

## Abstract

(1) Background: Recent economic developments in South Korea have shifted people’s interest in forests from provisioning to cultural services such as forest healing. Although policymakers have attempted to designate more forests for healing purposes, there are few established standards for carrying out such designations based on the quantified estimation. (2) Methods: We suggest a modeling approach to estimate and analyze the emission rate of human-beneficial terpenes. For this purpose, we adopted and modified the Model of Emissions of Gases and Aerosols from Nature (MEGAN), a commonly used biogenic volatile organic compounds (BVOCs) estimation model which was suitable for estimating the study site’s terpene emissions. We estimated the terpene emission rate for the whole year and analyzed the diurnal and seasonal patterns. (3) Results: The results from our model correspond well with other studies upon comparing temporal patterns and ranges of values. According to our study, the emission rate of terpenes varies significantly temporally and spatially. The model effectively predicted spatiotemporal patterns of terpene emission in the study site. (4) Conclusions: The modeling approach in our study is suitable for quantifying human-beneficial terpene emission and helping policymakers and forest managers plan the efficient therapeutic use of forests.

## 1. Introduction

People in South Korea have heavily relied on forest ecosystem services for a long time as the forest is the dominant land cover type in the country, covering approximately 63% of the entire national territory [1]. Traditionally, the provision of services such as timber, fuel wood, herbaceous plants, and wild animals was the primary way in which forests were utilized. However, the rapid economic growth in the country has shifted people’s interest in utilizing forests from the direct use of forest products to the indirect use of cultural services, such as for recreational and therapeutic purposes in pursuit of well-being [2]. To satisfy the growing demand for using forests for recreational and therapeutic purposes, the government has designated several mountainous forests as recreational and healing forests and managed them via various programs for physically and psychologically improving public health [2,3].

Forest bathing is one of the most popular forest activities in South Korea and Japan from which therapeutic benefits from forests are derived [4,5,6]. Although forest bathing includes various activities performed in forests, the primary purpose is to take advantage of the physiological and psychological benefits of the forest. These benefits might result from inhaling and absorbing human-beneficial biogenic volatile organic compounds (BVOCs), such as terpenes and terpenoids, through the olfactory system and the skin [4,6].

BVOCs from forests are various chemical compounds emitted from trees during metabolic processes and in the course of responding to various environmental changes [6,7,8]. Their effects, especially terpenes, on human health and welfare have already been well investigated by various studies (see the detailed reviews in [9,10]). Several studies have reported that terpenes can improve the immune system by activating human natural killer cells, which are helpful for eradicating tumors and virus-infected cells [4,11,12]. Terpenes also show anti-inflammatory [6,8,13], neuroprotective [14,15], anti-cancer [6,16], and relaxing effects [17].

As forest bathing is closely related to the terpenes emitted from the forest, understanding the spatiotemporal patterns of terpenes emission is essential for the effective management of forests for therapeutic use. Many studies have investigated the seasonal patterns of the emission of total BVOCs or specific compounds including terpenes from specific tree species [18,19,20,21], identifying biotic and abiotic factors affecting the emission of BVOCs from forests [22,23,24], and estimating the spatiotemporal patterns of regional BVOC emission [25,26,27,28]. However, in situ or experimental studies measuring BVOC emission from forests have focused on the ecological responses of individual tree species to specific environmental drivers in small plots, but they barely considered spatial patterns of BVOC emission. On the other hand, regional studies estimating BVOCs have evaluated the effect of BVOCs on atmospheric chemistry, including aerosols and ozone production, so they assessed BVOC emission at a coarse spatial resolution.

Although many in situ and experimental studies about BVOCs and terpene emissions in forests have been performed, only a few studies have attempted to quantify terpene emissions using a spatially explicit model suitable for assessing detailed therapeutic potential of forests. Meneguzzo et al. [29] measured total volatile organic compounds (TVOCs) to understand the variability of BVOC concentration in a forest, considering the spatial characteristics of the forest including hilly sites, forest paths, and hiking trails. At a high temporal resolution, they analyzed the temporal patterns of BVOC concentration during the day, which showed peaks during a few hours after noon, and in the morning. Similar studies have been conducted in South Korea to investigate the spatial and temporal patterns of BVOC concentration in forests. Park et al. [30] and Jeong et al. [31] sampled terpenes at several plots in therapeutic and recreational forests, focusing on the temporal variability of terpene concentration over the course of a day and by season. In addition, Choi et al. [32] characterized the effect of microclimatic factors on BVOC emission in bamboo groves located in an urban forest in South Korea by utilizing multilinear regression analysis. According to their study, BVOC emission from bamboo groves was positively correlated with the air temperature and humidity but negatively correlated with the wind and photosynthetically active radiation (PAR). Moreover, Lee et al. [33] investigated the characteristics of spatial and seasonal monoterpene emission patterns in recreational forests in Gyunggi Province, South Korea. They classified recreational forests into four categories, namely natural recreation forest, arboretum, the forest park, and city park. However, given the limitations of field measurements of previous studies, such as a limited number of samples, it is difficult to generalize the spatial and temporal characteristics of emissions and the concentration patterns of human-beneficial terpenes.

BVOC emission from trees is a complicated process which depends on meteorological factors such as temperature, solar radiation, soil water content, and atmospheric carbon dioxide (${\mathrm{CO}}_{2}$) concentration, as well as biological factors such as forest type and tree phenology [34,35,36]. As BVOC emission is a complex process determined by various factors, a spatially explicit simulation model is required to analyze the BVOC emission pattern spatially and temporally with a high resolution. The Model of Emissions of Gases and Aerosols from Nature (MEGAN) [34,35] is a commonly applied model for estimating the emission rate of volatile organic compounds originating from vegetation that considers plant functional types and species composition. The main purpose of this model was to estimate the emission of BVOCs in order to understand the effect of BVOCs on air quality, such as atmospheric aerosol and ozone formation [37]. By calculating the rate of emission of BVOCs for each compound class, including terpenes, the model can be feasibly used to estimate the emission rate of human-beneficial BVOCs from forests.

In this study, we aimed to propose a MEGAN-based model to understand the spatiotemporal patterns terpene emission and then suggest an optimal strategy for utilizing a recreational forest in South Korea. To achieve this goal, we adopted the MEGAN model and modified it to make it suitable for estimating human-beneficial terpene emission in a basin level. Furthermore, we then applied the model to a recreational forest in South Korea and assessed the model’s applicability for estimating the rate of terpene emission. We also analyzed the spatiotemporal characteristics of the rate of terpene emission in this recreational forest. Finally, we suggested optimal strategies for utilizing such forests for therapeutic purposes based on the spatiotemporal analysis.

## 2. Materials and Methods

### 2.1. Study Area

**Figure 1:**
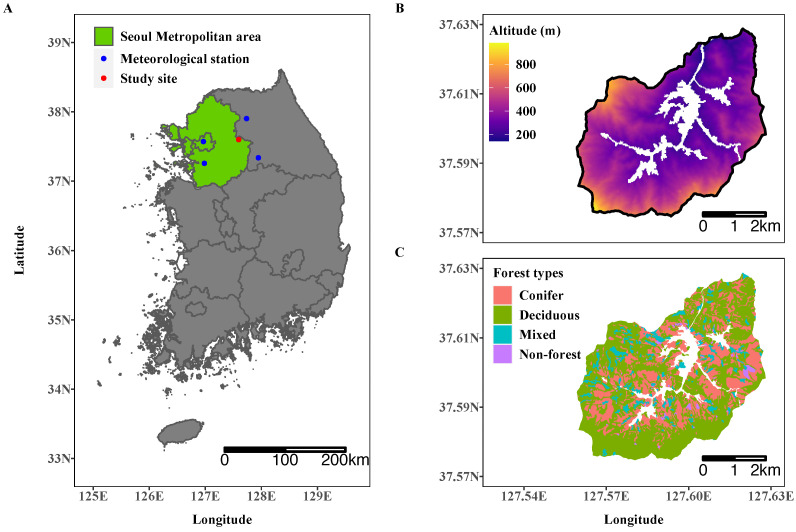
Location (**A**), topography (**B**), and forest types (**C**) of the study site (Saneum-ri). Blue points in (**A**) represent the meteorological stations near the study site providing solar irradiation data.

This study was performed in Saneum-ri, Gyunggi Province, where Saneum recreational forest, the largest national recreational forest in the Seoul metropolitan area, is located (Figure 1A). The total area of the site is approximately 2376 ha and the dominant land cover type is forest (93.6%), followed by agricultural land (5.2%), streams (0.6%), residential areas (0.4%), and roads (0.2%). The study site is a basin surrounded by Mt. Bongmi to the north, and Mt. Yongmun, Mt. Danwol, and Mt. Sori to the south (Figure 1B). Its altitude is in the range of 194 m to 954 m. The lower flat area of the study site is mostly used as residential and agricultural land and for roads. Forests cover the upper part of the study site with steep slopes, and are mainly used for recreational purposes such as hiking and forest bathing. In the study site, the forested land is composed mostly of deciduous (61.7%) followed by conifer (31.0%) and mixed (6.4%) forests (Figure 1C).

### 2.2. Model of Emissions of Gases and Aerosols from Nature (MEGAN)

The MEGAN model [34] was developed as a tool for assessing the effect of the atmospheric chemistry of natural ecosystems on global climate change and regional air quality by estimating the rate of emission of biogenic volatile organic compounds (BVOCs) [34,35]. Initially, the model only calculated the emission rate of isoprene, which is the dominant BVOC and often generates ozone and aerosols through chemical reactions with other atmospheric contaminants such as nitrogen oxides (NOx) [34]. The updated version, MEGAN v2.1 [35], can additionally estimate the emission rates of 18 compound classes, including 11 human-beneficial terpenes.

The MEGAN v2.1 model determines the rate of emission of each BVOC species from plants, considering the plant functional type (PFT), environmental conditions (i.e., light, temperature, soil water, and atmospheric carbon dioxide (${\mathrm{CO}}_{2}$) concentration), as well as plants’ source density and physiological activity [35]. The rate of emission of a chemical compound *i* of a region ($F_{i}$: $\mu g\;m^{-2}\;h^{-1}$) containing *k* PFTs can be estimated as

(1) $$F_{i}\;=\;\gamma_{i}\;\sum_{j=1}^{k}\varepsilon_{i,j}\;\chi_{j}.$$

Here, $\gamma_{i}$ is the emission activity factor of the chemical compound *i*, $\varepsilon_{i,j}$ is the emission rate of the chemical compound *i* of the PFT *j* under standard conditions (see Table 1), and $\chi_{j}$ is the cover ratio of the PFT *j* in a region.

The emission activity factor ($\gamma_{i}$) represents the relative emission rate considering given environmental and plant physiological conditions compared with the rate under standard conditions, which can be described as

(2) $$\gamma_{i}\;=\;C_{\mathrm{CE}}\;\cdot \;{\mathrm{LAI}}_{v}\;\cdot \;\gamma_{P,i}\;\cdot \;\gamma_{T,i}\;\cdot \;\gamma_{A,i}\;\cdot \;\gamma_{SM,i}\;\cdot \;\gamma_{C,i},$$

where $C_{\mathrm{CE}}$ is the canopy environment coefficient, which makes $\gamma_{i}$ unity under standard conditions. In addition, $\gamma_{P,i}$, $\gamma_{T,i}$, $\gamma_{SM,i}$, and $\gamma_{C,i}$ are the emission factors accounting for the response to the environmental conditions of light, temperature, soil moisture, and atmospheric ${\mathrm{CO}}_{2}$, respectively. ${\mathrm{LAI}}_{v}$ and $\gamma_{A,i}$ are the emission factors corresponding to the plant physiological conditions described by the effective leaf area index (LAI) and leaf age. In the MEGAN model, the effective LAI (${\mathrm{LAI}}_{v}$) considers not only the leaf area index (LAI) from remote sensing data but also the vegetation cover ratio ($F_{v}$) and can be described as

(3) $${\mathrm{LAI}}_{v}\;=\;\frac{\mathrm{LAI}}{F_{v}}$$

Under standard conditions, a plant has an ${\mathrm{LAI}}_{v}$ value of 5 and has a canopy leaf-age composition of 80% mature, 10% growing, and 10% old leaves. In terms of environmental factors, the standard conditions refer to the solar angle of 60${}^{\circ }$, an atmospheric light transmittance of photosynthetically active radiation (PAR) of 0.6, air temperature of 303 K, humidity of 14 g k$g^{-1}$, wind speed of 3 m $s^{-1}$, and average temperature and photosynthetic photon flux density (PPFD) of the past 24 h to 240 h of 297 K and 200 μ$\mathrm{mol}\;m^{-2}\;s^{-1}$ for sun-exposed leaves (50 μ$\mathrm{mol}\;m^{-2}\;s^{-1}$ for shaded leaves), respectively, [35].

#### 2.2.1. Light-Responsive Emission Activity Factor ($\gamma_{P}$)

$\gamma_{P,i}$ is the light-responsive emission activity factor of BVOC species *i*, which is determined by the light-dependent fraction of BVOC species *i* (${\mathrm{LDF}}_{i}$), PPFD, average PPFD over the past 24 h ($P_{24}$) and 240 h ($P_{240}$). The LDF values for each BVOC species account for the different emission activities of each compound species corresponding to given light conditions (see Table 2).

The equation for $\gamma_{P,i}$ can be represented as follows:

(4) $$\gamma_{P,i}\;=\;\left(1-{\mathrm{LDF}}_{i}\right)\;+\;{\mathrm{LDF}}_{i}\;\cdot \;\gamma_{P_{LDF}}.$$

$\gamma_{P_{LDF}}$ is the light-dependent light-responsive activity factor determined by $C_{p}$, α, and PPFD, which is described as

(5) $$\gamma_{P_{LDF}}\;=\;C_{p}\;\cdot \;\frac{\alpha \;\cdot \;\mathrm{PPFD}}{\sqrt{1\;+\;{\left(\alpha \;\cdot \;\mathrm{PPFD}\right)}^{2}}}.$$

α and $C_{p}$ are defined as below:

(6) $$\begin{array}{cc} \alpha & \;=\;0.004-0.005\;\cdot \;\ln P_{240}, \end{array}$$

(7) $$\begin{array}{cc} C_{p} & \;=\;0.0468\;\cdot \;{P_{240}}^{0.6}\;\cdot \;e^{0.0005\;\cdot \;(P_{24}-P_{S})} \end{array}$$

where $P_{S}$ is the PPFD under standard conditions.

#### 2.2.2. Temperature-Responsive Emission Activity Factor ($\gamma_{T}$)

$\gamma_{T,i}$ is the temperature-responsive emission activity factor of BVOC species *i*, which is determined by LDF, leaf temperature (*T*), and the average leaf temperature over the past 24 h ($T_{24}$) and 240 h ($T_{240}$). The equation for $\gamma_{T,i}$ can be described as

(8) $$\gamma_{T,i}\;=\;\left(1\;-\;{\mathrm{LDF}}_{i}\right)\;\cdot \;\gamma_{T_{LIF},i}\;+\;{\mathrm{LDF}}_{i}\;\cdot \;\gamma_{T_{LDF},i}$$

where $\gamma_{T_{LIF},i}$ and $\gamma_{T_{LDF},i}$ are the light-independent and light-dependent temperature-responsive activity factors, respectively. $\gamma_{T_{LDF},i}$ is determined by compound-species-specific empirical coefficients (i.e., $C_{eo,i}$ and $C_{T1,i}$, which are described in Table 2) considering the difference between average leaf temperatures (i.e., $T_{24}$ and $T_{240}$) and the temperature under standard conditions ($T_{S}$), which can be described as

(9) $$\gamma_{T_{LDF},i}\;=\;\frac{230\;\cdot \;C_{eo,i}\;\cdot \;e^{0.05\;\cdot \;\left(T_{240}+T_{24}\;-\;2\;\cdot \;T_{S}\right)\;+\;C_{T1,i}\;\cdot \;x}}{230\;-\;C_{T1,i}\;\cdot \;\left(1-e^{230\;\cdot \;x}\right)}$$

where *x* is defined as follows

(10) $$x\;=\;\frac{1}{0.0083}\;\cdot \;\left(\frac{1}{313\;+\;0.6\;\cdot \;(T_{240}-T_{S})}-\frac{1}{T}\right).$$

$\gamma_{T_{LDF},i}$ is determined by *T* and $T_{S}$ with compound-species-specific empirical coefficient $\beta_{i}$ (see Table 2).

(11) $$\gamma_{T_{LDF},i}\;=\;e^{\beta_{i}\;\cdot \;\left(T-T_{s}\right)}$$

#### 2.2.3. Leaf-Age Emission Activity Factor ($\gamma_{A}$)

$\gamma_{A,i}$ is the factor determining the emission activity of BVOC compound species *i* by the plant’s leaf-age composition, which is the product of the fraction of leaves by their age class and the empirical compound-species-specific coefficients corresponding to each leaf-age class. The equation for $\gamma_{A,i}$ is

(12) $$\gamma_{A,i}\;=\;F_{new}\;\cdot \;A_{new,i}\;+\;F_{gro}\;\cdot \;A_{gro,i}\;+\;F_{mat}\;\cdot \;A_{mat,i}\;+\;F_{sen}\;\cdot \;A_{sen,i}$$

where $F_{new}$, $F_{gro}$, $F_{mat}$, and $F_{sen}$ represent the fractions of new, growing, mature, and senescent leaves and $A_{new}$, $A_{gro}$, $A_{mat}$, and $A_{sen}$ represent the empirical compound-species-specific coefficients of new, growing, mature, and senescent leaves, respectively, which are shown in Table 2. $\gamma_{A,i}$ for evergreen conifer trees is assumed to have a value of 1 due to them maintaining their leaves year-round [34]. For deciduous trees, the fraction of leaves by their age class can be estimated through the change in LAI over time [34]. In cases when the current LAI (${\mathrm{LAI}}_{c}$) is equal to the LAI of the previous time step (${\mathrm{LAI}}_{p}$), $F_{new}$, $F_{gro}$, $F_{mat}$, and $F_{sen}$ are set to be 0, 0.1, 0.8, and 0.1, respectively. In cases where ${\mathrm{LAI}}_{p}$ is greater than ${\mathrm{LAI}}_{c}$, $F_{new}$, $F_{gro}$, $F_{mat}$, and $F_{sen}$ are equal to 0, 0, ${\mathrm{LAI}}_{c}/{\mathrm{LAI}}_{p}$, and $\left({\mathrm{LAI}}_{p}-{\mathrm{LAI}}_{c}\right)/{\mathrm{LAI}}_{p}$, respectively. In the other cases of ${\mathrm{LAI}}_{c}$ being greater than ${\mathrm{LAI}}_{p}$, $F_{sen}$ is equal to 0 and other fractions adhere to the following rules:

(13) $$\begin{array}{c} \begin{array}{cc} F_{new} & =\left\{\begin{array}{cc} 1\;-\;\frac{{\mathrm{LAI}}_{p}}{{\mathrm{LAI}}_{c}} & \mathrm{for}\;t\;\leq \;t_{i}\\ \frac{t_{i}}{t}\;\cdot \;\left(1-\frac{{\mathrm{LAI}}_{p}}{{\mathrm{LAI}}_{c}}\right) & \mathrm{for}\;t\;>\;t_{i} \end{array}\right.\\ F_{mat} & =\left\{\begin{array}{cc} \frac{{\mathrm{LAI}}_{p}}{{\mathrm{LAI}}_{c}} & \mathrm{for}\;t\;\leq \;t_{m}\\ \frac{{\mathrm{LAI}}_{p}}{{\mathrm{LAI}}_{c}}\;+\;\frac{t\;-\;t_{m}}{t}\;\cdot \;\left(1-\frac{{\mathrm{LAI}}_{p}}{{\mathrm{LAI}}_{c}}\right) & \mathrm{for}\;t\;>\;t_{m} \end{array}\right.\\ F_{gro} & =1\;-\;F_{new}\;-\;F_{mat} \end{array} \end{array}$$

where *t* is the time interval of the LAI database, which is usually obtained from periodically revisited remote sensing data. $t_{i}$ is the number of days between the bud break and induction of BVOC emission, which depends on the average air temperature of the preceding time step interval ($T_{t}$). $t_{m}$ is the number of days between bud break and the initiation of the peak BVOC emission rate, which has a strong correlation with $t_{i}$.

(14) $$\begin{array}{cc} t_{i} & =\left\{\begin{array}{cc} 5\;+\;0.7\;\cdot \;(300\;-\;T_{t}) & \mathrm{for}\;T_{t}\;\leq \;303\\ 2.9 & \mathrm{for}\;T_{t}\;>\;303 \end{array}\right. \end{array}$$

(15) $$\begin{array}{cc} t_{m} & =2.3\;\cdot \;t_{i} \end{array}$$

#### 2.2.4. Soil Moisture and ${\mathrm{CO}}_{2}$-Responsive Emission Activity Factors ($\gamma_{SM}$ and $\gamma_{C}$)

As soil moisture and atmospheric ${\mathrm{CO}}_{2}$ concentration only affect the emission of isoprene, the soil moisture ($\gamma_{SM,i}$) and the atmospheric ${\mathrm{CO}}_{2}$ ($\gamma_{C,i}$)-responsive emission activity factors of all compound species other than isoprene are set to be 1 in the MEGAN v2.1 model. $\gamma_{SM,isoprene}$ is determined by the volumetric soil moisture content (θ: $m^{3}\;m^{-3}$) of a region, which is estimated as

(16) $$\begin{array}{cc} \gamma_{SM,isoprene} & =\left\{\begin{array}{cc} 1 & \mathrm{for}\;\theta \;>\;\theta_{1}\\ \frac{\theta \;-\;\theta_{w}}{\Delta \theta_{1}} & \mathrm{for}\;\theta_{w}\;<\;\theta \;<\;\theta_{1}\\ 0 & \mathrm{for}\;\theta \;<\;\theta_{w} \end{array}\right. \end{array}$$

where $\theta_{w}$ is the wilting point, $\theta_{1}$ is the minimum soil water content sufficient for the emission of isoprene, and $\Delta \theta_{1}$ is the difference between $\theta_{1}$ and $\theta_{w}$ ($\Delta \theta_{1}=\theta_{1}\;-\;\theta_{w}$) with an empirical value of 0.06. $\gamma_{C,isoprene}$ is estimated as

(17) $$\gamma_{C,isoprene}=I_{S_{max}}\;\cdot \;\left(1\;-\;\frac{C_{i}^{h}}{C_{*}^{h}\;+\;C_{i}^{h}}\right)$$

where $I_{S_{max}}$, $C_{*}$, and *h* are empirical coefficients determined by atmospheric ${\mathrm{CO}}_{2}$ concentration level and $C_{i}$ is the 70 % of the ambient ${\mathrm{CO}}_{2}$ level [35,38].

### 2.3. MEGAN Model Setup

Estimating the rates of emission of human-beneficial terpenes with the MEGAN model requires hourly measured meteorological data (i.e., leaf and air temperature and PPFD), PFTs, and the effective LAI (${\mathrm{LAI}}_{v}$) [34,35]. We obtained hourly measured temperature determined at 10 m above the ground from the mountain meteorology observation system installed at Mt. Bongmi for ambient air temperature and leaf temperature of the site [39]. PPFD was calculated utilizing data on the average hourly solar radiation ($MJ\;m^{-2}$) obtained from four weather stations [40] surrounding the study site. We applied the factor 277.78 to convert the hourly solar radiation from $MJ\;m^{-2}$ to $W\;m^{-2}$ and then applied 2.02 μ$\mathrm{mol}\;W^{-1}\;s^{-1}$ to convert solar radiation to PPFD [41,42]. We obtained PFT from the forest map provided by the Korea Forest Service [43] and also estimated LAI values from the normalized difference vegetation index (NDVI) using the empirical equation suggested by Jang et al. [44], which is adequate for South Korea.

(18) $$\begin{array}{cc} \mathrm{LAI} & =\;6.7537\times \mathrm{NDVI}+0.8384 \end{array}$$

(19) $$\begin{array}{cc} \mathrm{NDVI} & =\;\frac{\rho_{8}\;-\;\rho_{4}}{\rho_{8}\;+\;\rho_{4}} \end{array}$$

where NDVI is the normalized difference vegetation index utilizing red ($\rho_{4}$) and near-infrared ($\rho_{8}$) bands from Sentinel-2 L2 Surface Reflectance data obtained from the Google Earth Engine [45]. We adjusted unreasonably low values during monsoon season in the Sentinel-2 NDVI data and filled missing values by applying the Hampel filter utilizing the “hampel” function implemented in “pracma” package [46] as well as by applying a double logistic filter utilizing the function “FitDoubleLogBeck” implemented in the “phenopix” package [47] in the statistical computing language R 4.0.5 [48]. The Hampel filter determines unusual points in time series data by comparing each point with neighboring points within a certain window size. The point is classified as an outlier when it is markedly different from neighboring points over a certain threshold and it is replaced with a corrected value [46,49]. Double logistic fitting is one of the widely used methods for smoothing phenological time series data usually obtained from satellite images [50,51,52]. The input parameters and their sources are summarized in Table 3.

### 2.4. MEGAN Model Modification and Implementation

In this study, we modified the model to enable an application at a finer spatial resolution of 30 m to better consider the site-specific meteorology and the detailed information on the vegetation and topography of the study area. To consider the effect of the topographic complexity on the light-dependent emission factor for calculating $\gamma_{P}$, we estimated the relative intensity of solar radiation by calculating the cosine of the angle between the direction of the sun and the direction normal to the surface using the R package “insol” [53,54]. To focus on estimating the rate of terpene emission, we only calculated three emission factors: $\gamma_{P}$, $\gamma_{T}$, and $\gamma_{A}$. On the other hand, $\gamma_{SM}$ and $\gamma_{C}$, which are only required for calculating the emission of isoprene, were set to 1.

We implemented the modified MEGAN model on R 4.0.5 based on the algorithm presented by Guenther et al. [34] and Guenther et al. [35], and source codes of the MEGAN v2.1 program. The original MEGAN model focuses on estimating the rates of emission of BVOCs on a coarse regional scale.

### 2.5. Analysis of Human-Beneficial Terpene Emission

To suggest the optimal use of forests for inhaling human-beneficial terpenes, we analyzed the temporal and spatial patterns of monoterpene and sesquiterpene emission utilizing R 4.0.5. To understand the temporal patterns of terpene emission from forests, we calculated the hourly, intra-day, and seasonal variations of their emission averaged across the whole study site. We classified three intra-day periods of morning, afternoon, and evening corresponding to time ranges from 08:00 to 10:00 h, from 14:00 to 16:00 h, and from 20:00 to 22:00 h, respectively, and compared the BVOCs and terpene emission rates of each period of the day using Kruskal–Wallis one-way analysis of variance. We also compared the seasonal terpene emission rates by classifying the year into spring, summer, fall, and winter, corresponding to March to May, June to August, September to November, and December to February, respectively.

For the spatial patterns of terpene emission in the study area, we calculated the annual mean rate of terpene emission to determine the spatial emission patterns for the whole study area. To investigate the effect of topography and plant function type (PFT), we compared the terpene emission rate by slope aspects and by PFT using Kruskal–Wallis one-way analysis of variance.

## 3. Results

### 3.1. Temporal Variation of Human-Beneficial Terpene Emission Rate

**Figure 2:**
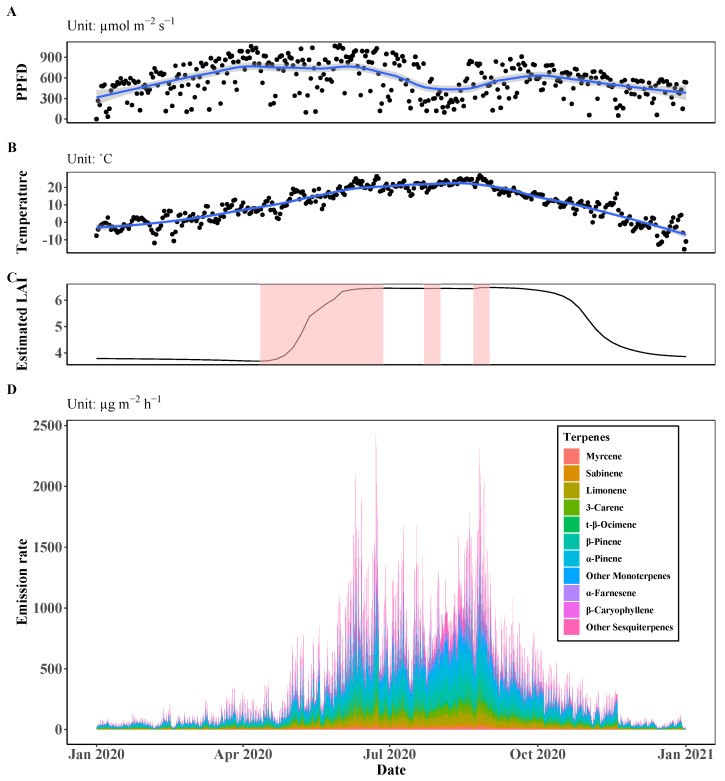
Temporal trends of the environmental and physiological input variables of the MEGAN model ((**A**) daily mean PPFD, (**B**) daily mean temperature, and (**C**) estimated LAI applying gap filling and smoothing) and the average terpene emission rate in Saneum-ri area in (**D**). Blue lines and shaded areas in (**A**,**B**) represent the smoothing lines and the 95% confidence interval using the locally estimated scatterplot smoothing (Loess) method. The red area in (**C**) represents the period with increased LAI.

We calculated the mean emission rate of each terpene in the Saneum-ri area during the whole year in 2020 with hour-long time windows (Figure 2).

The results show a higher overall emission rate during summer, covering from June to early September, but a lower level during winter. We identified the temporarily lowered emission rate in the rainy season during the summer monsoon period. The PPFD level was also low because cloud cover frequently blocked solar irradiation at that time.

The most emitted type of terpene in the study area was monoterpenes, which include compounds with high emission rates such as α-pinene (24.6%), β-pinene (21.8%), and limonene (9.6%) (see Table 4). In contrast, the proportion of sesquiterpene was relatively small.

**Figure 3:**
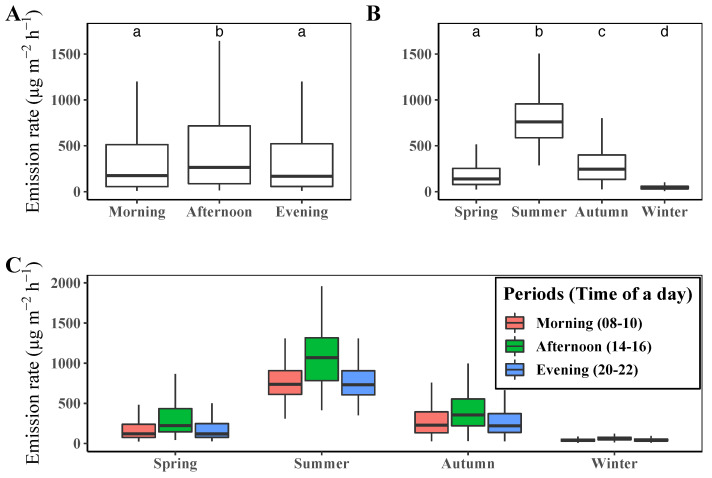
(**A**) Mean diurnal variation of terpene emission rate, (**B**) seasonal and (**C**) seasonal–diurnal comparison of the terpene emission rate of the year 2020 in Saneum-ri area. Different letters above the boxplots in (**A**,**B**) indicate significant differences (Dunn’s test *p* < 0.05) among mean values of terpene emission rate.

The diurnal and seasonal patterns of terpene emission are presented in Figure 3.

In terms of the diurnal variation of terpene emission rates, there was high variability among the different times of the day. Our model estimated the highest level of emission around the middle of the day and the lowest level around sunrise.

The terpene emission rates also significantly differed among the seasons. The average emission rate was the highest in summer, followed by autumn, spring, and winter. The diurnal variation of the terpene emission rate was more significant in summer, while little difference was observed in winter. We also calculated the seasonal mean emission rates of each compound class, as presented in Table 5.

Similar to the total emission rate of terpenes, the emission rate of each compound class varied with the season, with monoterpenes being dominant in all seasons. The emission rate of each terpene type (i.e., monoterpenes and sesquiterpenes) was highest in summer and lowest in winter. The ratios between the highest and lowest emission rates were 15.1 and 153.8 for monoterpenes and sesquiterpenes, respectively, indicating that the emission of monoterpenes was more stable than that of sesquiterpenes across the seasons.

### 3.2. Spatial Patterns of Terpene Emission Rate

**Figure 4:**
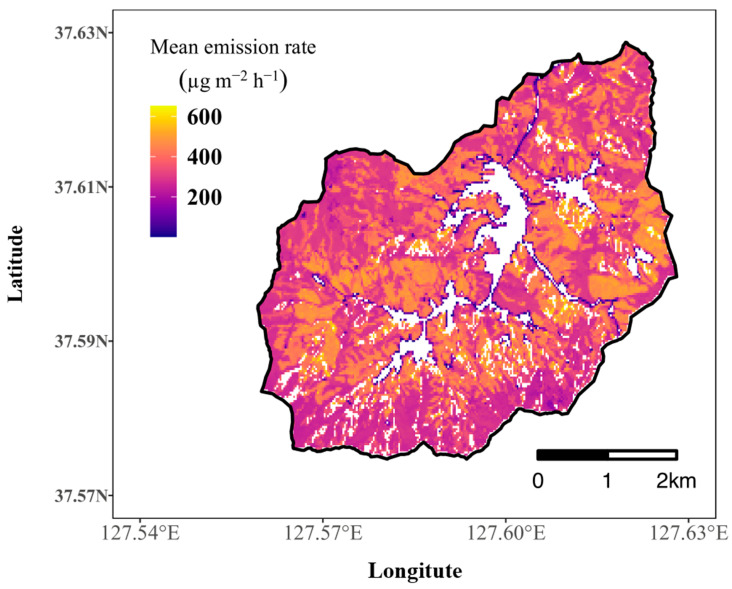
Spatial patterns of the terpene emission rate in Saneum-ri area in the year 2020.

We projected the model to the study area and represented the annual mean emission rate of total terpenes as a map (see Figure 4).

The average emission rate of the study area in the year 2020 was 347.2 μ$g\;m^{-2}\;h^{-1}$ ranging from 1.6 μ$g\;m^{-2}\;h^{-1}$ to 651.6 μ$g\;m^{-2}\;h^{-1}$. The terpene emission in the model is affected by both slope aspects through regulating solar radiation and the PFTs represented as the forest map in this study. However, the results demonstrate that the PFTs are more important for determining the amount of terpene emission rates in the study area. The resulting map shows the high spatial variations of the emission rate, and the spatial pattern appeared to match well with the forest type presented in Figure 1A. These results indicate that annual terpene emission is closely related to PFTs in this area.

### 3.3. Comparison of Terpene Emission Rate by Slope Aspect

**Figure 5:**
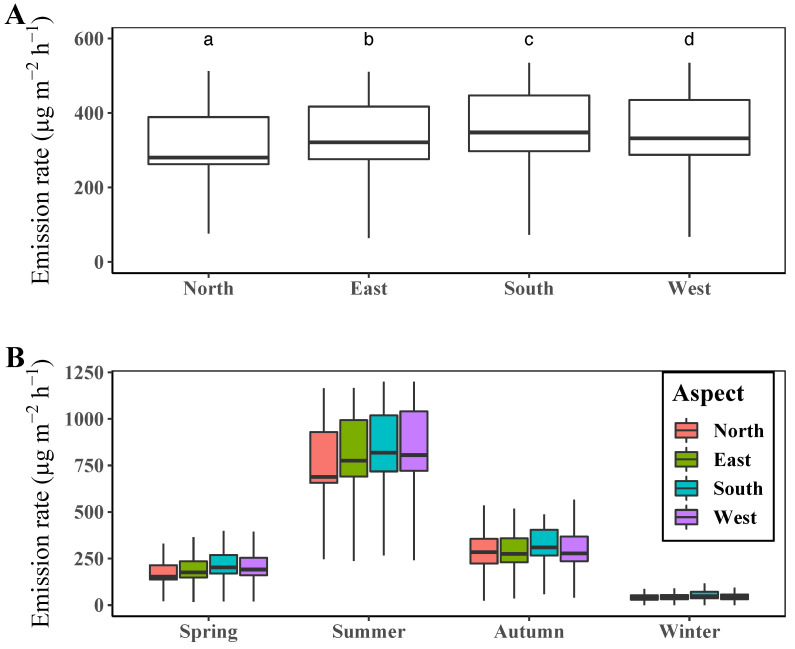
(**A**) Mean terpene emission rate by slope aspect during the whole year in 2020 and (**B**) for each season. Different letters above the boxplots in (**A**) indicate significant differences (Dunn’s test *p* < 0.05) among mean values of terpene emission rate.

We also compared the differences of terpene emission by slope aspect during a year and for each season (Figure 5).

The average terpene emission rate of a year differed significantly among the slope aspect groups (Figure 5A). The highest rates was estimated in south-facing slopes, while the lowest level was in north-facing ones. The emission rate was notably lower in north-facing slopes than in the other slopes. As the amount of solar irradiation is generally higher at south-facing slopes than at north-facing ones in the northern hemisphere, the emission level was also estimated to be higher at the south-facing slopes than at the north-facing ones. The emission rates by slope aspect for each season showed similar patterns to those during a year, except for a higher level being reported in the north-facing slopes than in the east- and west-facing ones during autumn (Figure 5B).

### 3.4. Comparison of Terpene Emission Rate by Plant Functional Type (PFT)

**Figure 6:**
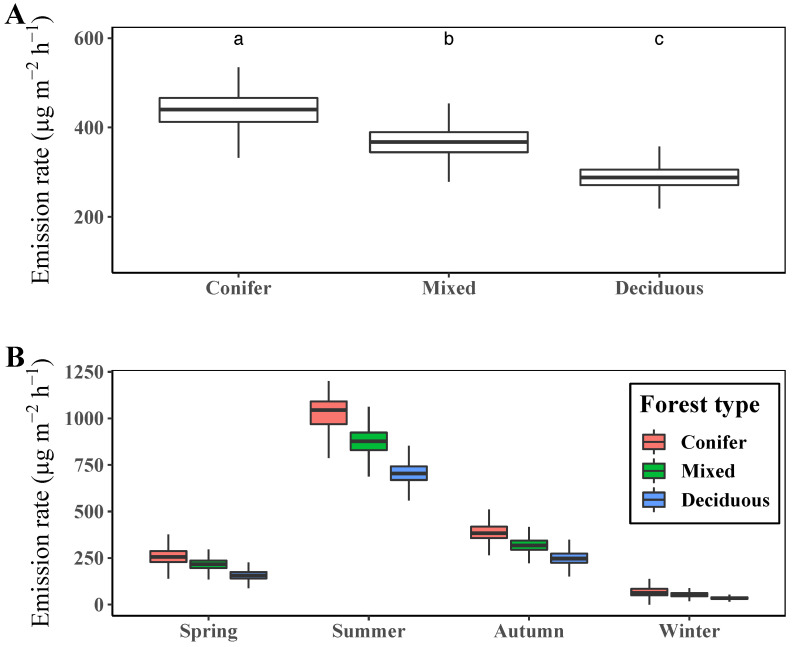
Mean terpene emission rate by plant functional type (PFT) for the whole year (**A**) and for each season (**B**). Different letters above the boxplots in **A** indicate significant differences (Dunn’s test *p* < 0.05) among mean values of terpene emission rate.

When we compared the annual mean rate of terpene emission of each PFT, conifer forest showed the highest level, while deciduous forest showed the lowest (Figure 6A).

The mixed forests showed a moderate emission level as they have conifer and deciduous trees at almost equal levels. The difference in the terpene emission level among PFTs was notable in the growing season, such as in summer, while this difference was negligible in winter when the plants stop growing (see Figure 6B).

## 4. Discussion

### 4.1. Spatiotemporal Patterns of Terpenes

In this study, we estimated the spatiotemporal characteristics of terpenes emitted from a recreational forest utilizing the concept of the MEGAN model. We found that the terpene emissions rate in the temperate forest where our study site is located varies substantially in time and space.

As shown in Figure 2B,D, the temporal trend of the emission of terpenes generally follows that of the temperature, which is consistent with the results of Laffineur et al. [55] which show that temperature is the main driver of terpene emission. The close relationship between the emission rate and temperature can also be found in the diurnal trend shown in Figure 3A. In this figure, we found that the peak emission rate was not in the middle of the day but at 2 or 3 h after that, when the surface temperature was highest after the surface of the land was heated by the sun. Although the general emission pattern follows the temperature, we also identified the importance of solar irradiance and the change of LAI for terpene emission. In the temporal trend shown in Figure 2D, we found a period with a temporarily lowered terpene emission level from late June to mid-August during summer, even though the temperature remained high. This period coincides with the summer rainy season when the solar radiance decreases abruptly because of the frequent development of thick cloud cover at the East Asian Monsoon front (Figure 2A) [56]. We also found that the terpene emission rate rapidly increased when LAI increased, as shown in the red areas in Figure 2C. This simultaneity indicates that plant growth can directly affect terpene emission since terpenes are by-products of plants’ metabolism.

Although both the diurnal and seasonal variations in terpene emission are significant, the long-term variation is more significant than the short-term variation. As shown in Figure 3A,B, the mean diurnal variation is in the range of 249.2 μ$g\;m^{-2}\;h^{-1}$ to 477.9 μ$g\;m^{-2}\;h^{-1}$ and the mean seasonal variation is in the range of 47.3 μ$g\;m^{-2}\;h^{-1}$ to 812.2 μ$g\;m^{-2}\;h^{-1}$, with the minimum value in winter and the maximum value in summer. The differences in a day and among seasons are approximately 228 μ$g\;m^{-2}\;h^{-1}$ and 765 μ$g\;m^{-2}\;h^{-1}$, respectively. We also found that the difference among periods of a day in each season is smaller than that among the seasons (Figure 3C). As terpene emission is affected by temperature, solar irradiance, and LAI, which change slowly in the short term but significantly in the phenological timescale, the seasonal change is more remarkable than the daily change. The physiological activity, including plant growth, is high when the temperature and solar irradiance are favorable, and the changes in LAI arise from the physiological activity determined by environmental factors. Therefore, the temporal variations of terpene and other environmental and physiological factors demonstrate strong relationships between terpene emission and plants’ physiological activity, which is consistent with the result of Llusia et al. [57].

According to the results, PFTs are the main factors determining the spatial pattern of terpene emissions, which matches well with previous studies [19,55]. In our study, slope aspects affected the site-specific incoming solar irradiance, and PFTs were closely related to the tree species, which have strong correlations with the composition and amount of terpene emissions [19,55,58]. Although the terpene emission rate differs significantly depending on the slope aspect and PFT, the differences among PFTs are more remarkable than those among slope aspects, as shown in Figure 5 and Figure 6. In terms of slope aspects, annual mean terpene emission was highest at south-facing slopes with 358 μ$g\;m^{-2}\;h^{-1}$ and lowest at north-facing slopes with 313 μ$g\;m^{-2}\;h^{-1}$, which differ by at most 45 μ$g\;m^{-2}\;h^{-1}$. The annual mean terpene emission rate by forest showed the highest level in the conifer forest (434 μ$g\;m^{-2}\;h^{-1}$) and the lowest level in the deciduous forest (291 μ$g\;m^{-2}\;h^{-1}$), with a maximum difference of 134 μ$g\;m^{-2}\;h^{-1}$. In addition, the spatial pattern of the mean annual terpene emission rate in Figure 4 resembles those of forest types in Figure 1. These results demonstrate that PFTs can discriminate the spatial patterns of the terpene emission rate in the study site. Moreover, the slight differences in the amount of incoming solar irradiance determined by slope aspects cannot significantly affect the spatial patterns of terpene emission.

### 4.2. Validity of the Model for Estimating Terpenes

In this study, we tested the validity of the MEGAN model for estimating terpenes by comparing model outputs with the measured and calculated values from other studies due to the lack of field measurement data to validate the model used in this study. According to our results, the seasonal pattern of the terpene emission rate was as follows: low in winter, early spring, and late fall, and high in summer and early fall from June to September. This matches well with the results from other studies. Sindelarova et al. [59] estimated global monoterpene emission and reported a high emission rate in summer from June to August with a peak in July and a very low emission rate in the other seasons of the year for temperate forests in the northern hemisphere. Cho et al. [60] calculated the emission rate of monoterpenes in the whole of South Korea and found higher emission rates from June to September and lower ones in winter. Similar patterns were found in field-based studies. Chen et al. [24] also showed the highest emission rates from June to September when they measured the monoterpene emission rate from five dominant tree species in northern China, based on the enclosure technique. In addition, Jeong et al. [31] demonstrated a high terpene concentration in Saneum recreational forest from July to September, with the terpene concentration being measured by an ambient air sampling method.

Our study showed a high terpene emission rate in the afternoon and lower emission levels at other times. Nonetheless, other studies have shown diverse diurnal trends of terpene emissions depending on the measurement methods and measuring objects to concern (i.e., flux or concentration). Studies focusing on the emission rate or flux of terpenes showed similar diurnal patterns as in Figure 3 [19,55,57,61,62,63]. In this case, the emission rate showed a strong dependence on the temperature and solar irradiance. On the other hand, studies that measured the terpene concentration in ambient air showed diverse diurnal patterns. Several studies that measured BVOC and terpene concentration in the ambient air showed diurnal patterns that were similar to ours [29,30], while other studies showed different diurnal patterns of high BVOC and terpene concentrations in the morning and before sunset and lower concentrations in the middle of the day [25,31,32,64]. Comparing the two types of results from other studies, the difference in diurnal terpene emission patterns between the two types of measurement scheme arises from the mixing effect related to wind speed and rapid chemical reactions with other materials in the air (e.g., ozone formation) [27,30,32,63,65].

Owing to the rate of emission of terpenes varying markedly in time and space and even among different measurement methods [65], there is limited value in attempting to validate the model by directly comparing the terpene emission rates from our study to those from other studies. Among studies with comparable results, Tani et al. [61] reported that the maximum α-pinene emission rate was observed in June, with a value of 490.4 μ$g\;m^{-2}\;h^{-1}$, which is similar to our result with a maximum value of 541.8 μ$g\;m^{-2}\;h^{-1}$ estimated in June. In addition, Laffineur et al. [55] reported monoterpene emission rates of approximately 972 μ $g\;m^{-2}\;h^{-1}$ and 334.8  μ$g\;m^{-2}\;h^{-1}$ during the daytime and nighttime in summer (from July to September), respectively, which can be averaged to approximately 653.4  μ$g\;m^{-2}\;h^{-1}$, which is consistent with our mean summer emission rate of monoterpenes ( 688.5 μ$g\;m^{-2}\;h^{-1}$). According to Nagori et al. [63], the measured and estimated monoterpene emission rates from June to early July ranged from approximately 200 μ$g\;m^{-2}\;h^{-1}$ to 1400 μ$g\;m^{-2}\;h^{-1}$ and from 400 μ$g\;m^{-2}\;h^{-1}$ to 1000 μ$g\;m^{-2}\;h^{-1}$, respectively, which is consistent with our result calculated during June (from 446.6 μ$g\;m^{-2}\;h^{-1}$ to 967.8 μ$g\;m^{-2}\;h^{-1}$).

Based on the comparison of our results with those from other studies, we can verify our methods in terms of temporal patterns and the range of values, which shows the feasibility of our approach utilizing MEGAN for estimating the level of terpene emission in our study site. Our method is based on the rate of emission of terpenes directly from trees, and the result differs from the concentration-based methods sampling ambient air. The therapeutic effect of forest bathing and inhaling terpenes is directly related to the terpene concentration in the ambient air, which is not only associated with the terpene emission rate from trees but also closely related to microclimatic factors, such as humidity and wind speed and direction [25,31,32,63,64]. Therefore, there is a need for a deeper understanding and consideration of the microclimate in order to estimate the spatiotemporal variation of terpenes directly affecting humans.

### 4.3. Optimal Strategies for Using Forests for Therapeutic Purposes

Our findings demonstrate that the emission rate of human-beneficial terpenes in our temperate forest study site varies significantly temporally and spatially, emphasizing the need for elaborate strategies to effectively use therapeutic forests. Our study also shows the usefulness of MEGAN models for establishing forest management plans for therapeutic use. Through estimating and analyzing the spatiotemporal patterns of terpene emission from the MEGAN model, we can suggest effective strategies for using therapeutic forests. Temporally, it is recommended that forests be used for therapeutic purposes in the middle of the day, around noon to sunset, to inhale the maximum level of terpenes there. In addition, it is encouraged to plan therapeutic programs in recreational and healing forests in summer, especially before and after the rainy season, at which the highest emission of terpenes occurs. Spatially, the most critical factor determining the pattern of terpene emission is PFTs, the forest types in our study, while the slope aspect also affects the emission level. Therefore, it is recommended that forest managers prepare space for therapeutic programs in conifer forests facing south in order to maximize the effectiveness of terpenes. Moreover, the planting of conifer trees can be effective when there is a lack of such trees in recreational and healing forests. As such, the MEGAN-based estimation of the emission of human-beneficial terpenes considering the climate and the forest type can help forest managers effectively utilize forests for therapeutic purposes.

## 5. Conclusions

We estimated and analyzed the terpene emission rate spatiotemporally in a recreational forest adjacent to the Seoul metropolitan area, South Korea, utilizing a MEGAN model. We found that the terpene emission rate in our study area varied significantly in time and space. When we analyzed the temporal variation of the terpene emission rate, the highest levels occurred during the middle of the day and seasonally during summer before and after the rainy season. Our model also showed the effect of plant functional types and slope aspect on the spatial patterns of the terpene emission rate. Our results especially emphasize the importance of PFTs, with remarkable differences in emission rates being identified among PFTs. According to the comparison of our results with those from other studies, estimating the rate of emission of human-beneficial terpenes using MEGAN appears feasible because the temporal patterns and range of human-beneficial terpene emission rates from our study are consistent with those from other studies. Using our results, we can more effectively manage forests for therapeutic purposes by planning therapeutic programs in optimal times and locations. Although we cannot consider microclimatic factors such as wind and humidity in our study, our work still demonstrates the usefulness of the MEGAN model for forest management for therapeutic use.

## Figures and Tables

**Table 1:** Emission rate ($\mu g\;m^{-2}\;h^{-1}$) of each BVOC species under standard conditions for PFTs in the temperate climate regions (adapted from Table 2 in Guenther et al. [35]).

Compound Class	Compound Species	Needleleaf Conifer	Broadleaf Deciduous
Isoprene	Isoprene	600	10,000
Monoterpene	Myrcene	70	30
	Sabinene	70	50
	Limonene	100	80
	3-Carene	160	30
	*t*-β-Ocimene	70	120
	β-Pinene	300	130
	α-Pinene	500	400
	Others	180	150
Sesquiterpene	α-Farnesene	40	40
	β-Caryophyllene	80	40
	Others	120	100
Other VOCs	232-MBO	700	0.01
	Methanol	900	900
	Acetone	240	240
	CO	600	600
	Bidirectional VOC	500	500
	Stress VOC	300	300
	Others	140	140

**Table 2:** Parameters for MEGAN model (adapted from Table 4 in Guenther et al. [35]).

Compound Class	Compound Species	β	LDF	$C_{t1}$	$C_{\mathrm{eo}}$	$A_{\mathrm{new}}$	$A_{\mathrm{gro}}$	$A_{\mathrm{mat}}$	$A_{\mathrm{old}}$
Isoprene	Isoprene	0.13	1.0	95	2.00	0.05	0.60	1.00	0.90
Monoterpene	Myrcene	0.10	0.6	80	1.83	2.00	1.80	1.00	1.05
	Sabinene	0.10	0.6	80	1.83	2.00	1.80	1.00	1.05
	Limonene	0.10	0.2	80	1.83	2.00	1.80	1.00	1.05
	3-Carene	0.10	0.2	80	1.83	2.00	1.80	1.00	1.05
	*t*-β-Ocimene	0.10	0.8	80	1.83	2.00	1.80	1.00	1.05
	β-Pinene	0.10	0.2	80	1.83	2.00	1.80	1.00	1.05
	α-Pinene	0.10	0.6	80	1.83	2.00	1.80	1.00	1.05
	Others	0.10	0.4	80	1.83	2.00	1.80	1.00	1.05
Sesquiterpene	α-Farnesene	0.17	0.5	130	2.37	0.40	0.60	1.00	0.95
	β-Caryophyllene	0.17	0.5	130	2.37	0.40	0.60	1.00	0.95
	Others	0.17	0.5	130	2.37	0.40	0.60	1.00	0.95
Other VOC	232-MBO	0.13	1.0	95	2.00	0.05	0.60	1.00	0.90
	Methanol	0.08	0.8	60	1.60	3.50	3.00	1.00	1.20
	Acetone	0.10	0.2	80	1.83	1.00	1.00	1.00	1.00
	CO	0.08	1.0	60	1.60	1.00	1.00	1.00	1.00
	Bidirectional VOC	0.13	0.8	95	2.00	1.00	1.00	1.00	1.00
	Stress VOC	0.10	0.8	80	1.83	1.00	1.00	1.00	1.00
	Others	0.10	0.2	80	1.83	1.00	1.00	1.00	1.00

**Table 3:** List of input parameters and their sources for the MEGAN model of the study site.

Parameter	Source
Air and leaf temperatures	Temperature measured 10 m above the ground at Mt. Bongmi from Korea Forest Service [39]
PPFD	Average solar radiation from four meteorological stations surrounding the site [40].
Plant functional type (PFT)	Forest type of forest map (1:5000) from Korea Forest Service [43]
Leaf area index (LAI)	Estimated from NDVI index derived from Sentinel-2 L2 surface reflectance data with a 10 m spatial resolution obtained from Google Earth Engine [45]

**Table 4:** Annual mean emission rate (μ$g\;m^{-2}\;h^{-1}$) of each terpene in Saneum-ri area.

Compound Class	Compound	Annual Mean Emission Rate (μg m${}^{-2}$h${}^{-1}$)	Proportion (%)
Monoterpene	α-Pinene	82.8	24.6
	β-Pinene	73.3	21.8
	Limonene	32.4	9.6
	3-Carene	30.6	9.1
	Sabinene	10.9	3.3
	Myrcene	8.7	2.6
	t-β-Ocimene	8.2	2.5
	Other Monoterpenes	46.1	13.7
	Total	292.9	87.1
Sesquiterpene	β-Caryophyllene	12.0	3.6
	α-Farnesene	8.5	2.5
	Other Sesquiterpenes	23.0	6.8
	Total	43.5	12.9

**Table 5:** Seasonal mean emission rate (μ$g\;m^{-2}\;h^{-1}$) of each compound class of terpenes in the Saneum-ri area.

Season	Compound Class	Mean Emission Rate (μg m${}^{-2}$h${}^{-1}$)	Proportion (%)
Spring	Monoterpene	175.6 ± 140.6	90.6
	Sesquiterpene	18.1 ± 23.9	9.4
Summer	Monoterpene	688.5 ± 239.7	84.8
	Sesquiterpene	123.9 ± 74.0	15.2
Autumn	Monoterpene	259.3 ± 172.2	89.8
	Sesquiterpene	29.5 ± 29.4	10.2
Winter	Monoterpene	45.5 ± 22.6	96.2
	Sesquiterpene	1.8 ± 1.6	3.8

## Data Availability

Not applicable.
